# Unique Pattern of Component Gene Disruption in the NRF2 Inhibitor KEAP1/CUL3/RBX1 E3-Ubiquitin Ligase Complex in Serous Ovarian Cancer

**DOI:** 10.1155/2014/159459

**Published:** 2014-07-09

**Authors:** Victor D. Martinez, Emily A. Vucic, Kelsie L. Thu, Larissa A. Pikor, Roland Hubaux, Wan L. Lam

**Affiliations:** BC Cancer Research Centre, BC Cancer Agency, 675 West 10th Avenue, Vancouver, BC, Canada V5Z 1L3

## Abstract

The NFE2-related factor 2 (NRF2) pathway is critical to initiate responses to oxidative stress; however, constitutive activation occurs in different cancer types, including serous ovarian carcinomas (OVCA). The KEAP1/CUL3/RBX1 E3-ubiquitin ligase complex is a regulator of NRF2 levels. Hence, we investigated the DNA-level mechanisms affecting these genes in OVCA. DNA copy-number loss (CNL), promoter hypermethylation, mRNA expression, and sequence mutation for *KEAP1*, *CUL3*, and *RBX1* were assessed in a cohort of 568 OVCA from The Cancer Genome Atlas. Almost 90% of cases exhibited loss-of-function alterations in any components of the NRF2 inhibitory complex. CNL is the most prominent mechanism of component disruption, with RBX1 being the most frequently disrupted component. These alterations were associated with reduced mRNA expression of complex components, and NRF2 target gene expression was positively enriched in 90% of samples harboring altered complex components. Disruption occurs through a unique DNA-level alteration pattern in OVCA. We conclude that a remarkably high frequency of DNA and mRNA alterations affects components of the KEAP1/CUL3/RBX1 complex, through a unique pattern of genetic mechanisms. Together, these results suggest a key role for the KEAP1/CUL3/RBX1 complex and NRF2 pathway deregulation in OVCA.

## 1. Introduction

Reactive oxygen species (ROS) participate in normal hormonogenesis and physiological functions of the ovaries, such as steroid hormone production, ovulation, and essential preovulatory responses [1–3]. Hence, tight regulation of ROS levels in the ovaries is required.

**Figure 1:**
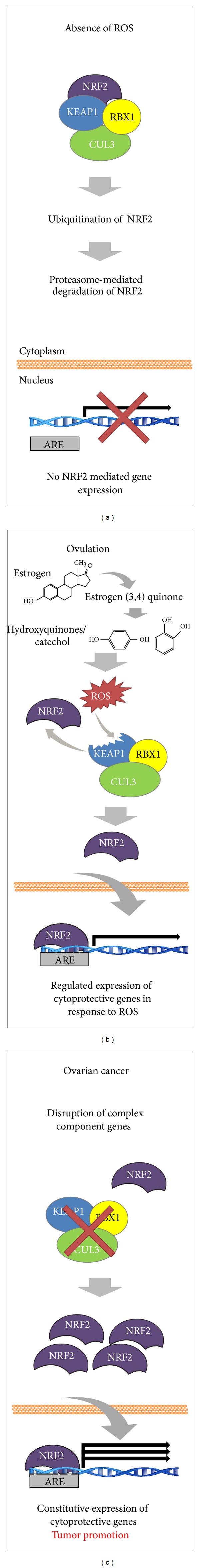
*KEAP1/CUL3/RBX1 E3-ligase protein complex*. In the absence of ROS (a), NRF2 is regulated by the KEAP1/CUL3/RBX1 E3-ubiquitin ligase complex which targets NRF2 for proteasomal degradation and inhibits expression of NRF2-controlled genes. The oxidative metabolism of estrogen through the catechol pathway induces the generation of reactive oxygen species (ROS, b). These oxidative species induce conformational changes in KEAP1, which disrupt the activity of the inhibitory complex. As a consequence, NRF2 is stabilized and translocates to the nucleus, where it induces expression of cytoprotective genes containing NRF2-regulatory sequence motifs (e.g., antioxidant response elements, AREs). When the KEAP1/CUL3/RBX1 E3-ubiquitin ligase complex is compromised by genetic alteration in any of its component genes (c), NRF2 is stabilized and accumulated and transported to the nucleus. Under these conditions, the activation of cytoprotective genes becomes constitutive, which has been associated with tumor promotion.

The NFE2-related factor 2 (NRF2) pathway is the primary regulator of cellular ROS levels (reviewed in [4–7]). Under basal conditions, NRF2 protein—encoded by the* NFE2L2* gene—is rapidly targeted for proteasomal degradation through interaction with an E3-ubiquitin ligase protein complex, whose protein components include Kelch-like ECH-associated protein 1 (KEAP1), Cullin 3 (CUL3), and ring-box 1, E3-ubiquitin protein ligase (RBX1) (Figure 1). KEAP1 acts as a substrate adaptor, interacting with NRF2 through ETGE and extended DLG motifs [8, 9]. Subsequently, NRF2 interacts with the CUL3 N terminal region, while RBX1 recruits the catalytic function of ubiquitin-conjugating enzyme (E3) [10]. An abnormal increase in ROS levels induces the formation of disulfide bonds between cysteine residues of KEAP1, which liberates NRF2, although some studies have suggested that electrophilic modification of Keap1 does not lead to complex disruption [11, 12]. Moreover, a cyclic degradation model involving sequential binding of NRF2 first to the ETGE motif and then through the DLG motif has been proposed [13]. This allows its translocation to the nucleus and subsequent induction of cytoprotective genes [6, 14, 15].

Besides its protective role, an emerging concept is that constitutive activation of NRF2 and its target genes can result in promotion of tumor growth and resistance to oxidants and anticancer drugs in a number of tumor types [6, 16, 17]. Constitutive activation of NRF2 is associated with acquisition of malignant features and has been demonstrated in various tumor types, including serous ovarian carcinoma (OVCA) [4, 18]. Gain-of-function mutations in* NFE2L2* and inactivating* KEAP1* mutations are the most frequent NRF2 activation mechanisms observed in breast, gallbladder, and lung tumors, among other cancer types [19–23]. Notably, multiple inactivating genetic mechanisms affecting components of the KEAP1/CUL3/RBX1 inhibitory complex are also known to occur, and the disruption of even a single complex component has been shown to compromise its function and stimulate substrate accumulation in lung tumors [24].

Traditional approaches for identifying driver alterations usually focus on high frequency, single-gene disruption. However, this approach may overlook biologically significant events, for example, when multiple gene products are required for proper multiprotein complex function [24–26]. For instance, a single component of a multiprotein complex or pathway may be disrupted at low frequency, but a high cumulative frequency of functional disruption may occur when alterations to individual complex components are simultaneously considered.

Genetic and epigenetic mechanisms underlying NRF2 activation in OVCA remain to be elucidated. A previous study identified heterozygous missense* KEAP1* mutations in 5 of 27 (19%) ovarian carcinomas, although frequencies differ across subtypes (29% and 8% in clear cell and serous tumors, resp. [27]). Interestingly, the same study noted 50% of tumors without* KEAP1* mutations exhibited nuclear localization of NRF2 protein (denoting pathway activation), suggesting that other mechanisms are likely driving NRF2 pathway activation in ovarian tumors. We hypothesized that DNA-level disruptions affecting the master NRF2 inhibitory complex may account for this discrepancy. Therefore, we assessed different types of DNA-level inactivating alterations (DNA sequence mutation, copy-number loss, and DNA hypermethylation) affecting the component genes of the CUL3/KEAP1/RBX1 E3-ubiquitin ligase complex in 568 OVCA cases from The Cancer Genome Atlas (TCGA) project.

## 2. Materials and Methods

### 2.1. Tumor Samples and Data Analysis

**Figure 2:**
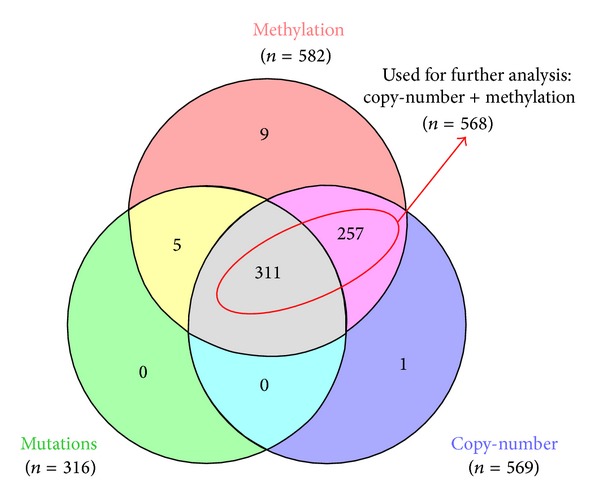
*Number of samples with the various types of data*. Information for DNA sequence mutation (green, *n* = 316), copy-number (purple, *n* = 569), and methylation (orange, *n* = 582) were retrieved from the cBio portal for Cancer Genomics. For subsequent frequency calculations comparing genetic and epigenetic mechanisms, we focused on the cases with both copy-number and methylation data (*n* = 568, i.e., cases circled in red).

Genomic and epigenomic information for OVCA were obtained from TCGA data portal (https://tcga-data.nci.nih.gov/tcga/) [28, 29] and the cBio portal for Cancer Genomics [30]. Level 3 data for DNA sequence mutation (somatic mutation calls for each participant), copy-number (putative copy-number calls, per sample), methylation (calculated beta values mapped to the genome, per sample), and mRNA (expression calls for genes, per sample) were used for analysis of different ‘omics dimensions (Figure 2).

### 2.2. DNA Sequence Mutations

Mutation data (derived from exome sequencing) were obtained for 316 cases (Figure 2). Mutation status and predicted functional impact was assessed through the cBioPortal for Cancer Genomics [31]. Nonsynonymous DNA sequence mutations with medium/high predicted functional impact scores were considered.

### 2.3. DNA Copy-Number Alterations

A total of 569 DNA copy-number profiles (Affymetrix GenomeWide SNP 6.0 platform) were obtained (Figure 2). In addition, copy-number data generated by the GISTIC algorithm [32] were also obtained through the cBio portal [31]. Both heterozygous (-1) and homozygous (-2) copy-number losses were considered when assessing inactivating DNA-level alterations affecting OVCA cases.

### 2.4. DNA Methylation Status

Methylation profiles (Illumina BeadArray 27K platform) for 582 samples were obtained from TCGA (Figure 2). Additionally, 8 profiles derived from organ-specific controls for ovarian tissue were retrieved for comparisons. Beta values (from probes located at promoter regions for each gene) were compared with beta values derived from organ specific controls. Differences (tumor-normal) ≥0.15 were considered hypermethylated in tumors.

### 2.5. mRNA Expression Profiling

Affymetrix U133 microarray and RNA sequencing data for* KEAP1*,* CUL3*, and* RBX1* mRNA expression were obtained from the cBio portal and TCGA data portal, respectively. Data from the Affymetrix U133 microarray (*n* = 370) were used for comparisons, since the number of samples with data available was higher than those available with RNA sequencing data for genes of interest.

### 2.6. Normalization of Expression Levels

In order to perform comparisons across the sample set, expression values were rank-normalized, in order to preserve the ordering of genes in a sample while removing any other factor affecting the set. For this, we used the “RankNormalize” package available through GenePattern [33].

### 2.7. Gene Set Enrichment Analysis

These analyses were performed using a single-sample gene set enrichment analysis (ssGSEA) [34]. Using rank-normalized expression levels, ssGSEA calculates separate enrichment scores (ES) that represent the degree to which each gene in a gene set is coordinately up- or downregulated within a sample. For enrichment analysis, we used 3 published gene sets (SINGH_NFE2L2_TARGETS, BIOCARTA_ARENRF2_PATHWAY, and V$NRF2_Q4) that contain genes either altered upon inactivation of NRF2 or genes that contain the NRF2 recognition motif (NTGCTGAGTCAKN) in the vicinity of its transcription start site [±2 kb].

### 2.8. Statistical Analysis

A comparison of the distributions of mRNA expression levels between samples with and without DNA-inactivating alterations in genes encoding complex components was performed in GraphPad Prism 6 (La Jolla, CA) using a Mann-Whitney *U* test. This analysis compared the differences in the median expression values between groups (no alterations versus any alterations) with 95% confidence.

### 2.9. Comparison of E3-Ubiquitin Ligase Complex Component Disruption across Different Tumor Types

To investigate whether patterns of disruption to the KEAP1/CUL3/RBX1 E3-ubiquitin ligase complex were specific to ovarian cancer, we assessed the frequency of genomic alterations in additional TCGA tumor types with (1) the largest number of samples with available multidimensional data through cBio portal and (2) data status indicating “No restrictions; all data available without limitations.” These included breast invasive carcinoma (BRCA), kidney renal clear cell carcinoma (KIRC), glioblastoma multiforme (GBM), lung adenocarcinoma (LUAD), lung squamous cell carcinoma (LUSC), head and neck squamous cell carcinoma (HNSC), thyroid carcinoma (THCA), and uterine corpus endometrial carcinoma (UCEC).

## 3. Results

### 3.1. CUL3/KEAP1/RBX1 E3-Ubiquitin Ligase Complex Is Frequently Disrupted by Multiple DNA Mechanisms in OVCA

First we investigated the frequency of DNA-level alterations (i.e., sequence mutations, copy-number loss, and hypermethylation) affecting each component gene of the CUL3/KEAP1/RBX1 E3-ubiquitin ligase complex. The results are summarized in Table 1. The disruption status of individual samples considering the various data types is shown in Figure 2.

**Figure 3:**
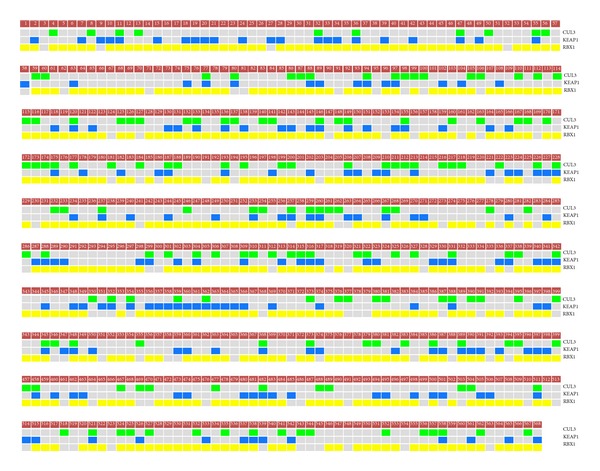
*DNA-level alteration affecting components of the KEAP1/CUL3/RBX1 E3-ubiquitin ligase complex in OVCA tumors*. Alteration status of individual complex component genes (*KEAP1* in blue,* CUL3* in green, and* RBX1* in yellow) across a panel of 588 ovarian tumors is indicated by colored boxes.

**Figure 4:**
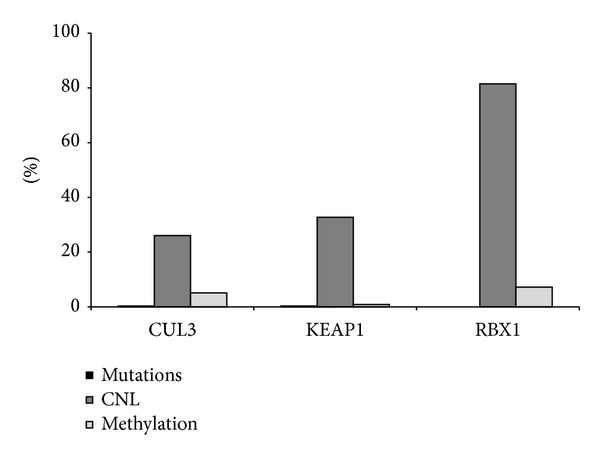
*Loss of function alterations affecting each complex component gene*. The frequency of DNA sequence mutations (black), DNA copy-number loss (CNL, dark grey), and promoter methylation (light grey) affecting each complex gene is shown.

Copy-number loss (CNL) was by far the most prominent inactivating mechanism affecting all complex components. Deletion of* CUL3*,* KEAP1*, and* RBX1* was detected in 26.0%, 32.7%, and 81.5% of samples, respectively (Table 1). Aberrant DNA methylation also affected component genes but at a much lower frequency than CNL. Somatic DNA mutations with significant predicted effects on protein function (according to Mutation Assessor) were found in only 2 samples (Table 1). Due to the low number of cases harboring mutations, we decided to focus our analysis on 568 samples with both copy-number and methylation data (Figure 3). Remarkably, when CNL and hypermethylation were considered concurrently, 90.5% of the OVCA cases examined sustained one or more alterations affecting any of the three components of the CUL3/KEAP1/RBX1 E3-ubiquitin ligase complex (Figure 3). The frequencies of individual alteration mechanisms were different among complex component genes (Figure 4).

### 3.2. DNA Alterations Affect Complex Component Gene Expression

**Figure 5:**
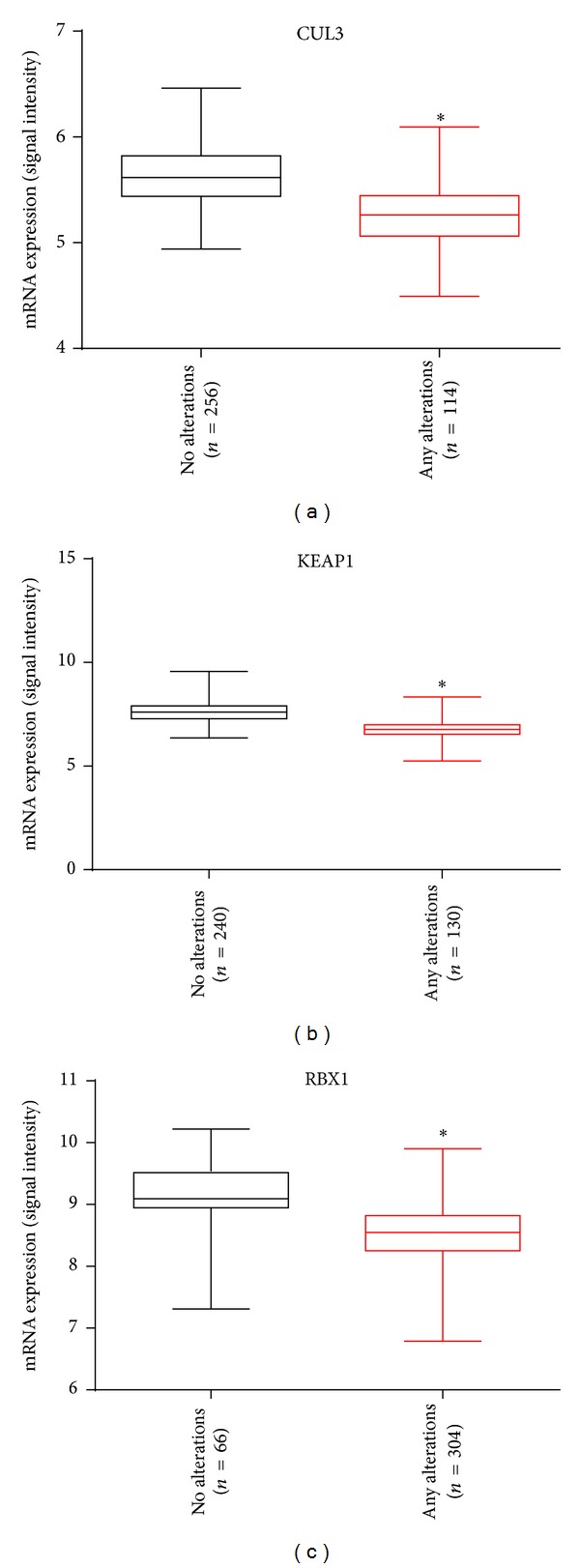
*Impact of DNA-level alteration on mRNA expression levels*. mRNA levels (measured as normalized array signal intensity) between OVCA groups with (black) and without (red) DNA-level alteration(s) were compared. ^∗^ indicates statistically significant differences (*P* < 0.01), assessed through the Mann-Whitney test.

We next evaluated the impact of DNA-level alterations on mRNA expression of* CUL3*,* KEAP1*, and* RBX1*, by comparing mRNA levels in samples with and without inactivating DNA-level alterations affecting any of the complex component genes for samples with available expression data for these genes (*n* = 37) (Figure 5). For the* CUL3*,* KEAP1*, and* RBX1* genes, mRNA levels were lower among samples harboring inactivating DNA-level alterations compared to those lacking these alterations (*P* value <0.01, Mann Whitney test), the vast majority of which were copy-number loss (Table 1).

### 3.3. Activation of NRF2 Target Genes Is Apparent in Samples Harboring DNA-Level Disruption of Complex Components

**Figure 6:**
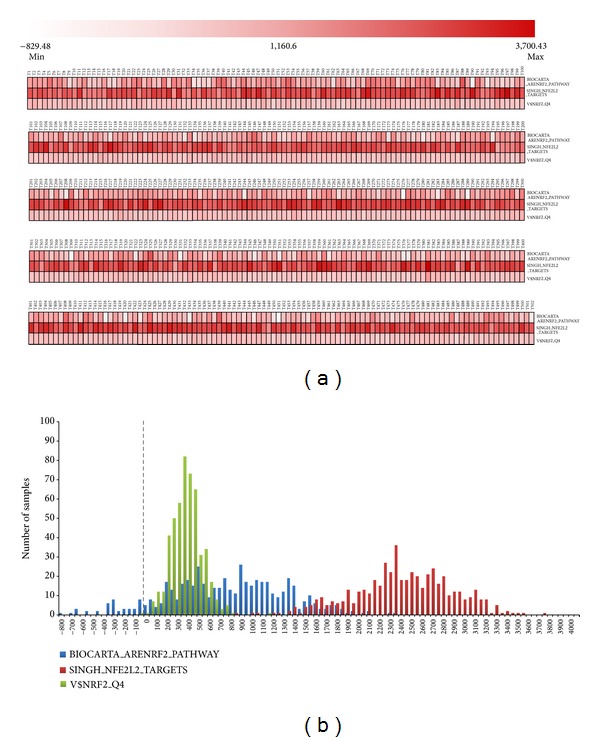
*Enrichment of NRF2 target genes in samples harboring complex component gene alteration*. (a) Enrichment of the different NRF2 target gene sets (Section 2). Enrichment scores (ES) are depicted for each sample. Increasing shades of red denote a larger ES. White boxes denote negative enrichment of NRF2 target genes (ES < 0). (b) Histogram for ES values across 502 samples with alterations affecting complex components genes.

We assessed activation of NRF2 target genes in each sample harboring DNA-level disruption affecting any component of the KEAP1/CUL3/RBX1 E3-ubiquitin ligase complex (*N* = 502) using single-sample gene set enrichment analysis (ssGSEA). ssGSEA assessed whether NRF2 gene sets were enriched in genes expressed in individual tumor samples (based on ranked gene expression levels within a tumor) (Figure 6(a)). Results for three different datasets from the Molecular Signatures Database revealed that 90.2% of samples harboring DNA-level alterations in complex components exhibited a positive enrichment for NRF2 target genes (Figure 6(b)).

### 3.4. OVCA Displays a Unique Pattern of NRF2 Inhibitory Complex Gene Disruption

**Figure 7:**
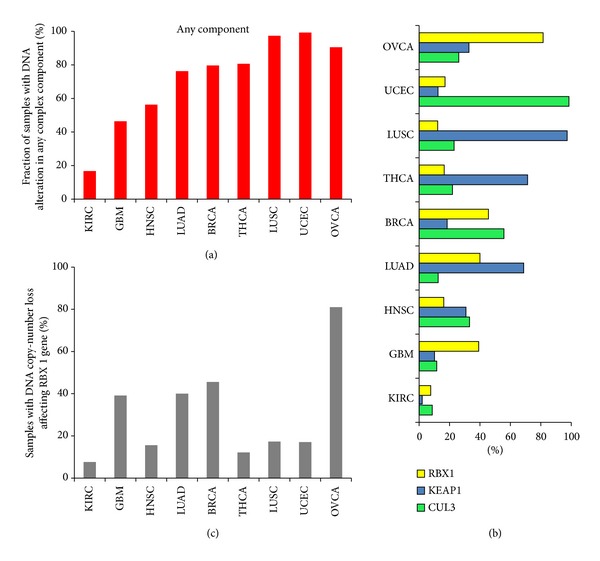
*Pan-cancer comparison of DNA alteration frequency affecting components of the KEAP1/CUL3/RBX1 E3-Ubiquitin ligase complex*. Frequency of DNA-level disruption (inactivating mutation, CNL, or hypermethylation) in ovarian carcinomas (OVCA) was compared to breast invasive carcinoma (BRCA), kidney renal clear cell carcinoma (KIRC), glioblastoma multiforme (GBM), lung adenocarcinoma (LUAD), lung squamous cell carcinoma (LUSC), head and neck squamous cell carcinoma (HNSC), thyroid carcinoma (THCA), and uterine corpus endometrial carcinoma (UCEC). (a) Proportion of tumors with 1 or more complex component genes disrupted by inactivating DNA-level mechanisms. (b) Frequency of disruption of individual complex component genes. (c) Frequencies of DNA copy-number loss (CNL) affecting the* RBX1* gene across multiple tumor types.

Given the importance of NRF2 activation in other malignancies, we next sought to determine how the spectrum of alterations in OVCA compares to other tumor types. We evaluated the frequency of* CUL3*,* RBX1*, and* KEAP1* disruption across multiple tumor types from the TCGA, selected based on data availability from TCGA (Section 2). Intriguingly, the frequency of complex disruption differed considerably across tumor types (Figure 7(a)), with an extremely high frequency of disruption in lung, thyroid, uterine, and ovarian tumors. Our analysis on individual complex component genes revealed that, in addition to different frequencies of disruption, each tumor type displays a distinctive pattern of CUL3/KEAP1/RBX1 E3-ubiquitin ligase complex alterations (Figure 7(b)). Overall,* KEAP1* (range: 2%–97.3%) and* CUL3* (range: 8.6%–98.4%) were typically the most frequently disrupted complex components. However, the frequency of alterations affecting* RBX1* in OVCA was the highest of any complex component gene in any of the tumor types analyzed, with CNL of* RBX1* observed in 81.5% of 568 cases (Figure 7(c)).

## 4. Discussion

Given the role of the NRF2 pathway in regulating cellular response to ROS, this pathway is likely critical to normal physiological ovarian function. However, the low reported frequency of inactivating* KEAP1* mutations does not account for the reportedly high frequency of NRF2 protein activation in ovarian cancer [18, 27, 35]. In this study, we provide evidence that inactivating genetic alterations affect multiple components of the CUL3/KEAP1/RBX1 E3-ubiquitin ligase NRF2 inhibitory complex in a remarkably high number of OVCA cases. These events are associated with a concordant reduction in component mRNA expression levels and positive enrichment of NRF2 target gene expression. Moreover, we note that OVCA sustains a unique pattern of complex component gene disruption compared to other cancer types, including those for which NRF2 activation through complex disruption are well known.

DNA-level inactivating alterations affecting gene components of the CUL3/KEAP1/RBX1 E3-ubiquitin ligase NRF2 inhibitory complex resulting in reduction of mRNA expression levels has been previously shown in thyroid, head and neck, and non-small cell lung tumors [24, 36, 37]. Moreover, these alterations were associated with a consequential increase in activated forms of complex ligands [24]. Concordant with our findings, we also observed a positive enrichment of NRF2 target genes in ~90% of OVCA samples harboring alteration in any of the individual complex component genes. This provides evidence of the potential effect of complex disruption on this pathway in disrupted tumors.

Interestingly, we did not observe DNA sequence mutations in the* NFE2L2 or KEAP1* genes in OVCA, even though this mechanism of NRF2 activation is well established in many tumor types [19–23]. This is consistent with the low frequency of* KEAP1* mutations observed in serous ovarian tumors in a previous study with a much smaller cohort [27]. Likewise, only 48 of the 568 samples (8.45%) exhibited segmental amplification and concurrent overexpression of* NFE2L2* suggesting other genetic mechanisms contribute to NRF2 protein and pathway activation in OVCA.

Analysis of the frequency of KEAP1/CUL3/RBX1 E3-ubiquitin ligase complex component gene disruption in a broad spectrum of cancer types revealed that component gene alteration is a common phenomenon in cancer, albeit at varying frequencies, suggesting this NRF2 inhibitory complex is important to many cancer types (Figure 7(a)). The frequency of disruption for ovarian tumors was comparable to the high frequencies observed in uterine carcinoma and lung squamous cell carcinoma (LUSC), where disruption of the KEAP1/CUL3/RBX1 E3-ubiquitin ligase complex is well established. Given that NRF2 inhibitory complex alterations are known to drive NRF2 pathway activation, we speculate that the alterations we have identified may contribute to the high frequency of aberrant NRF2 activation reported in ovarian cancer [18].

We found that* RBX1* sustained an extremely high frequency of copy-number loss, representing a characteristic of NRF2 inhibitory complex component disruption unique to OVCA.* RBX1* was altered in 81.5% of the OVCA tumors analyzed, compared to 26.05% and 32.74% for* CUL3* and* KEAP1*, respectively (Figure 7(b)). After OVCA, the highest frequency of CNL affecting* RBX1* was observed in breast cancer (BRCA), at 45.5%, while other gynecological tumors, such as uterine corpus endometrial carcinoma (UCEC), showed* RBX1* CNL in only 17.05% of cases (Figure 7(c)). Of note, the frequency of complex disruption in OVCA was similar to that seen in thyroid carcinoma (THCA) (Figure 7(a)), another organ that requires ROS for normal physiological function, hormonogenesis, and proliferation [36, 38, 39]. Taken together, these results demonstrate that the frequencies and patterns of alteration affecting KEAP1/CUL3/RBX1 E3-ubiquitin ligase complex components are tumor-type and tissue specific and that, in OVCA, copy-number loss affecting* RBX1* is the most prominent mechanism likely contributing to the increased NRF2 activation observed in ovarian cancer.

Given the extensive role of CUL3/KEAP1/RBX1 complex component proteins in other cellular pathways and functions, biological consequences of disruption to these genes certainly extend beyond the NRF2 pathway. For example, somatic disruption of KEAP1/CUL3 E3-ubiquitin ligase complex components also promote activation of NF-*κ*B in lung cancer, by compromising degradation of the NF-*κ*B activator, IKBKB [24]; given the extensive functions of NF-*κ*B, this may have broad implications to a multitude of biological systems of particular relevance to cancer. Moreover, KEAP1 interacts with other “ETGE” containing proteins that may be affected by KEAP1 disruption. Dipeptidyl peptidase 3 (DPP3), an ETGE containing protein, competes with endogenous NRF2 for binding to KEAP1 and is able to activate NRF2-mediated transcription [40]. Thus, it is plausible that DNA-level inactivation of KEAP1 may result in activation of DPP3 and subsequently pose an alternative pathway for NRF2 target gene activation. CUL3 can assemble with numerous substrate receptors with N-terminal BTB domains to form ubiquitin ligases complexes [41], whereby a shared catalytic core is able to recruit a variety of substrates (reviewed in [42, 43]). RBX1 is also a component of the von Hippel-Lindau (VHL) tumor suppressor complex, which interacts with Elongin B, Elongin C, and CUL2 [44]. Taken together, the potential implications of DNA-level alterations affecting components of the CUL3/KEAP1/RBX1 protein complex are broad and, cumulatively, may have profound implications in tumor biology.

While the frequency of DNA and mRNA level disruption we observed for KEAP1-CUL3-RBX1 complex components and correlation of these events with association NRF2 target gene transcriptional activation in ovarian cancer is compelling, we also acknowledge that other mechanisms might also impact NRF2 levels. For example, NRF2 activity can be repressed through another ubiquitin protein ligase complex, composed of beta-transducin repeat containing E3-ubiquitin protein ligase (BTRC), Cullin 1 (CUL1), and S-phase kinase-associated protein 1 (SKP1) [45, 46]. NRF2 is phosphorylated by GSK3, creating a phosphodegron to which BTRC is recruited [45]. To assess the possibility that alteration to these components may be contributing to NRF2 activation in ovarian tumors, we evaluated DNA-level alterations affecting the genes involved in this BTRC/SKP1/CUL1 complex. Interestingly, a high proportion of cases (84%) exhibited DNA-level alteration affecting at least one of the complex components. BTRC, GSK3A, and SKP1 exhibited a high frequency of DNA-level disruption, with 42%, 43%, and 45% of samples showing DNA copy-number losses, respectively. A significant effect on gene expression was also observed when samples with any alterations affecting BTRC, GSK3A, or SKP1 were compared against those cases without alterations affecting these complex components (see supplementary Figure 1 in Supplementary Material available online at http://dx.doi.org/10.1155/2014/159459). While the magnitude and frequency of KEAP1/CUL3/RBX1 complex component disruption were more prominent in the cohort we assessed, these results reveal the potential importance of alternative mechanisms of NRF2 activation in ovarian cancer and warrant consideration in future studies.

One of the main limitations of this study is the lack of protein level and/or localization analysis of complex components and NRF2 in OVCA tissues to confirm the biological effects of the DNA and mRNA level alterations we have described. We have addressed this by assessing enrichment of NFR2 target genes on each sample (ssGSEA), which has been previously used in OVCA cases from the TCGA project as well as in other studies [34, 47]. Use of this approach is especially relevant for cases where no clinical tissue specimens are available for immunohistochemical or similar analyses.

In conclusion, we have identified an extremely high frequency of genetic disruption affecting the KEAP1/CUL3/RBX1 E3-ubiquitin ligase complex in serous ovarian tumors, occurring predominantly through copy-number loss of* RBX1*. Disruption was associated with NRF2 pathway activation in the same individual tumors harboring complex alterations. Our observations highlight a potential mechanism underlying activation of NRF2 protein in OVCA. The high frequency of DNA-level complex disruption provides evidence that such disruption is selected in OVCA and further emphasizes the importance of NRF2 activation in this tumor type. Therapeutic targeting of NRF2 may represent a promising intervention point for serous ovarian tumor therapy; however, an improved understanding of the biological role of NRF2 in the context of ovarian tumor and nonmalignant (i.e., normal) cells must first be achieved, especially considering the importance of this pathway to normal ovarian function.

## Supplementary Material

Supplementary Figure 1: Alterations in the BTRC/SKP1/CUL1 complex A) DNA level alterations affecting genes involved in this BTRC/SKP1/CUL1 complex. We evaluated promoter hypermethylation (black), copy number losses (dark grey), and mutations (light grey) affecting BTRC, CUL1, GSK3A, GSK3B, and SKP1, as components of an alternative protein complex that might regulate NRF2 levels B) effects of DNA level alterations in the expression of GSK3A and SKP1 genes. Samples with no alteration (black) were compared with those exhibiting any alteration in the corresponding gene (red). Statistical comparison was performed using the Mann-Whitney test (“∗” indicates a significant p-value).

## Figures and Tables

**Table 1:** Frequency of OVCA cases affected by individual genetic mechanisms.

Gene complex component	Sequence mutation (*n* = 316)	Copy-number loss (*n* = 569)	Hypermethylation (*n* = 582)
*CUL3 *	1 (0.3%)	148 (26.01%)	30 (5.15%)
*KEAP1 *	1 (0.3%)	186 (32.69%)	5 (0.86%)
*RBX1 *	0 (0%)	464 (81.54%)	41 (7.04%)
