# Supplementary figures and images for: Unique Pattern of Component Gene Disruption in the NRF2 Inhibitor KEAP1/CUL3/RBX1 E3-Ubiquitin Ligase Complex in Serous Ovarian Cancer
Source: Biomed Res Int. 2014 Jul 9;2014:159459. doi: 10.1155/2014/159459 (PMC4121105; doi:10.1155/2014/159459)

**A**

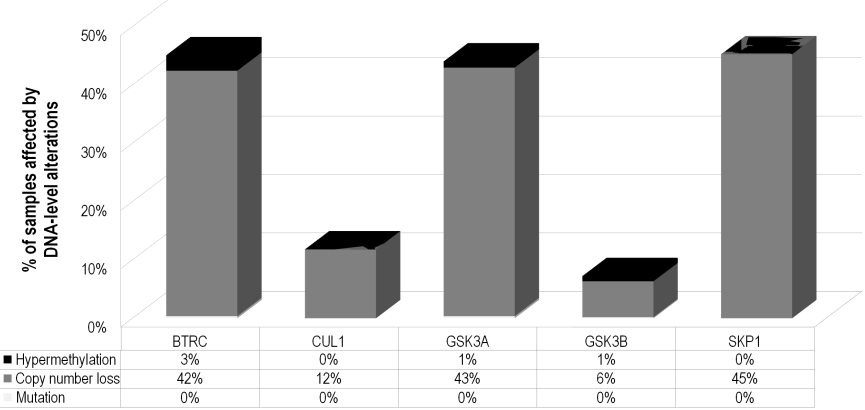

**B**

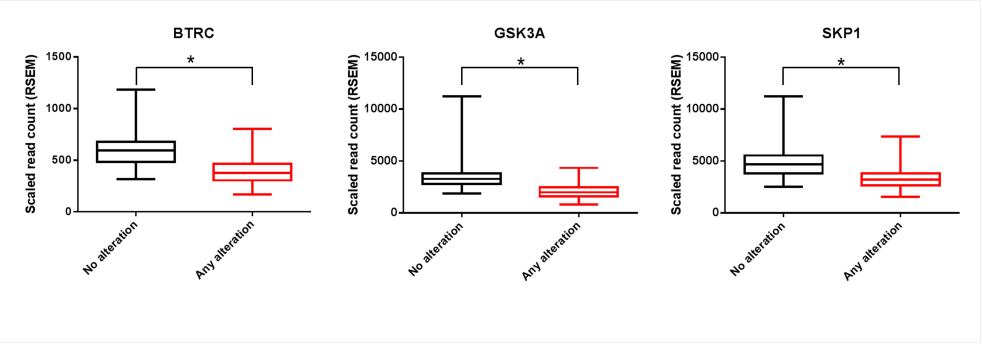

Supplement: Supplementary file 1 — Supplementary Figure 1: Alterations in the BTRC/SKP1/CUL1 complex A) DNA level alterations affecting genes involved in this BTRC/SKP1/CUL1 complex. We evaluated promoter hypermethylation (black), copy number losses (dark grey), and mutations (light grey) affecting BTRC, CUL1, GSK3A, GSK3B, and SKP1, as components of an alternative protein complex that might regulate NRF2 levels B) effects of DNA level alterations in the expression of GSK3A and SKP1 genes. Samples with no alteration (black) were compared with those exhibiting any alteration in the corresponding gene (red). Statistical comparison was performed using the Mann-Whitney test (“∗” indicates a significant p-value). [file 159459.f1.pdf]
